# Memory-guided navigation in amyotrophic lateral sclerosis

**DOI:** 10.1007/s00415-023-11753-8

**Published:** 2023-05-08

**Authors:** Patrizia M. Maier, Deetje Iggena, Thomas Meyer, Carsten Finke, Christoph J. Ploner

**Affiliations:** 1 Department of Neurology, Charité – Universitätsmedizin Berlin, Augustenburger Platz 1, 13353 Berlin, Germany; 2 Faculty of Philosophy, Berlin School of Mind and Brain, Humboldt-Universität zu Berlin, Berlin, Germany

**Keywords:** Navigation, Spatial memory, Hippocampus, Amyotrophic lateral sclerosis, Motor neuron disease

## Abstract

**Background:**

Previous studies have yielded inconsistent results about hippocampal involvement in non-demented patients with amyotrophic lateral sclerosis (ALS). We hypothesized that testing of memory-guided spatial navigation i.e., a highly hippocampus-dependent behaviour, might reveal behavioural correlates of hippocampal dysfunction in non-demented ALS patients.

**Methods:**

We conducted a prospective study of spatial cognition in 43 non-demented ALS outpatients (11f, 32 m, mean age 60.0 years, mean disease duration 27.0 months, mean ALSFRS-R score 40.0) and 43 healthy controls (14f, 29 m, mean age 57.0 years). Participants were tested with a virtual memory-guided navigation task derived from animal research (“starmaze”) that has previously been used in studies of hippocampal function. Participants were further tested with neuropsychological tests of visuospatial memory (SPART, 10/36 Spatial Recall Test), fluency (5PT, five-point test) and orientation (PTSOT, Perspective Taking/Spatial Orientation Test).

**Results:**

Patients successfully learned and navigated the starmaze from memory, both in conditions that forced memory of landmarks (success: patients 50.7%, controls 47.7%, *p* = 0.786) and memory of path sequences (success: patients 96.5%, controls 94.0%, *p* = 0.937). Measures of navigational efficacy (latency, path error and navigational uncertainty) did not differ between groups (*p* ≥ 0.546). Likewise, SPART, 5PT and PTSOT scores did not differ between groups (*p* ≥ 0.238).

**Conclusions:**

This study found no behavioural correlate for hippocampal dysfunction in non-demented ALS patients. These findings support the view that the individual cognitive phenotype of ALS may relate to distinct disease subtypes rather than being a variable expression of the same underlying condition.

**Supplementary Information:**

The online version contains supplementary material available at 10.1007/s00415-023-11753-8.

## Introduction

Amyotrophic lateral sclerosis (ALS) is a neurodegenerative disorder characterized by progressive degeneration of upper and/or lower motor neurons (MN) [1]. Although motor impairment is the most prominent symptom of ALS, a substantial proportion of patients also show non-motor deficits, including cognitive impairment [2]. Previous studies suggest that 13–20% of ALS patients develop frontotemporal dementia (FTD) [2–4]. Furthermore, ALS and FTD show overlap in genetic risk factors (e.g. C9orf72 repeat expansion), neuropathology (e.g. TDP-43 proteinopathy) and brain atrophy profiles [5, 6]. Additionally, cognitive and behavioural changes have been reported for non-demented ALS patients, with increasing prevalence in advanced disease stages [4, 7].

To date, the extent and nature of memory impairment in non-demented ALS patients is still unclear [8–11]. Structural abnormalities of the hippocampus have however been reported as a potential neural correlate for memory deficits in ALS. For example, imaging studies have reported hippocampal volume reductions that correlated with verbal memory performance in non-demented ALS patients [8, 10, 12]. Conversely, a recent study found no such alterations in non-demented ALS patients [5]. The absence of overt hippocampal dysfunction in studies of predominately motor-affected ALS patients may be due to limited sensitivity of behavioural tests [13], efficient compensation [14] or distinct neurodegenerative subtypes—thus challenging the view that ALS and FTD are variable phenotypes of the same underlying condition [5].

Here, we conducted a detailed study of spatial cognition in non-demented ALS-patients by using a memory-guided spatial navigation task. Studies in animals and humans have shown that spatial navigation critically depends on integrity of an extensive network that includes hippocampus, entorhinal cortex, parahippocampal cortex, retrosplenial cortex, prefrontal cortex and striatum [15–17]. Although dysfunction in any of these regions may yield deficits in navigational tasks, impaired spatial navigation has proven to be a particularly sensitive cognitive marker of hippocampal dysfunction in preclinical stages of neurodegenerative disorders such as Alzheimer’s disease [18]. We therefore reasoned that testing memory-guided navigation should reveal subtle or incipient hippocampal dysfunction in non-demented ALS patients. We employed a virtual maze task that has been used in animal and human studies of hippocampal function and that requires navigation to a target location based on memory of landmarks (‘allocentric’ condition) or memory of path sequences (‘egocentric’ condition) [19–21]. We further conducted neuropsychological tests of visuospatial memory, visuospatial fluency, and spatial orientation.

## Methods

### Participants

We investigated 43 patients, who were recruited from a specialised ALS centre and diagnosed according to the Gold Coast criteria for the diagnosis of ALS [22] (Table 1). Physical impairment and disease progression was measured with the ALS functional rating scale (ALSFRS-R) [23]. Thirty-two out of 43 patients were diagnosed with classic amyotrophic lateral sclerosis (upper and lower MN affected). Of these, twelve patients showed a bulbar onset, 20 patients a spinal onset. Four patients were diagnosed with primary lateral sclerosis (PLS, only upper MN signs) and seven with progressive muscular atrophy (PMA, only lower MN signs). Genetic testing was carried out in 20 patients (46.5%). In two patients, mutations in the FUS gene were detected, in one patient in the SOD1 gene and in one patient in the NEFH gene. All patients spoke German fluently, had normal or corrected-to-normal vision, normal hearing, denied substance abuse and had no neuropsychiatric disorders other than ALS. No cognitive deficits were reported by patients or their caregivers. No patient showed signs of ALS-FTD according to Strong criteria [24]. No patient fulfilled the Strong criteria for ALS with behavioral impairment (ALSbi) [24]. None of the patients showed signs of apathy with or without behavior change and no patient met two or more of the supportive diagnostic features for behavioral variant FTD [24, 25]. All patients showed sufficient hand motor function to control the joystick. In addition, 43 age-, sex- and education-matched healthy controls were tested (Table 1).

**Table 1:** Demographic and clinical data of patients and controls

	ALS (*n* = 43)	Control (*n* = 43)	*p* value
Female/male	11/32	14/29	0.63^1^
German native/non-native	41/2	41/2	1.00^1^
Age	60.0 (54.0–66.5)	57.0 (52.5–65.0)	0.23^2^
Years of education	15.0 (12.7–17.0)	16.0 (14.0–19.0)	0.08^2^
Self-rated spatial abilities (SBSDS)	4.8 (4.3–5.5)	5.0 (4.4–5.3)	0.97^2^
MN involvement (ALS/PLS/PMA)	32/4/7		
Months since initial symptoms	27.0 (16.0–52.0)		
Months since diagnosis	11.0 (6.0–28.0)		
ALSFRS-R	40.0 (35.5–43.0)		
ALSFRS-R progression/month	0.3 (0.1–0.4)		

Categorical data presented as absolute frequency. Continuous data presented as median and interquartile range (25–75%)

Abbreviations: *ALS* amyotrophic lateral sclerosis, *PLS* primary lateral sclerosis, *PMA* primary muscular atrophy, *SBSDS* Santa Barbara Sense of Direction scale, *FRS* functional rating scale

^1^*χ*^2^-test

^2^Wilcoxon rank sum test

### Neuropsychological assessment

All participants were tested with the German version of the *Edinburgh Cognitive and Behavioural ALS Screen (ECAS)* [26]. The ECAS is a cognitive assessment that is independent of motor disability and consists of 15 subtests across five domains: language, executive functioning, verbal fluency, verbal memory and visuospatial functioning. We compared the scores to age- and education-adjusted norm values. We further administered three neuropsychological tests of cognitive functions that relate to spatial navigation. Visuospatial fluency was assessed with the *Five Point Test (5PT)* [27]. Visuospatial memory was tested with the *10/36 Spatial Recall Test (SPART)* from the Repeatable Battery of Neuropsychological Tests (BRB-N) [28]. Spatial orientation was assessed with the *Perspective Taking/Spatial Orientation Test (PTSOT)* [29]. In addition, all participants rated their spatial abilities, preferences and experiences by completing the German version of the *Santa Barbara Sense of Direction scale (SBSOD)* [30].

### Behavioural assessment

#### Virtual navigation setup

**Fig. 1:**
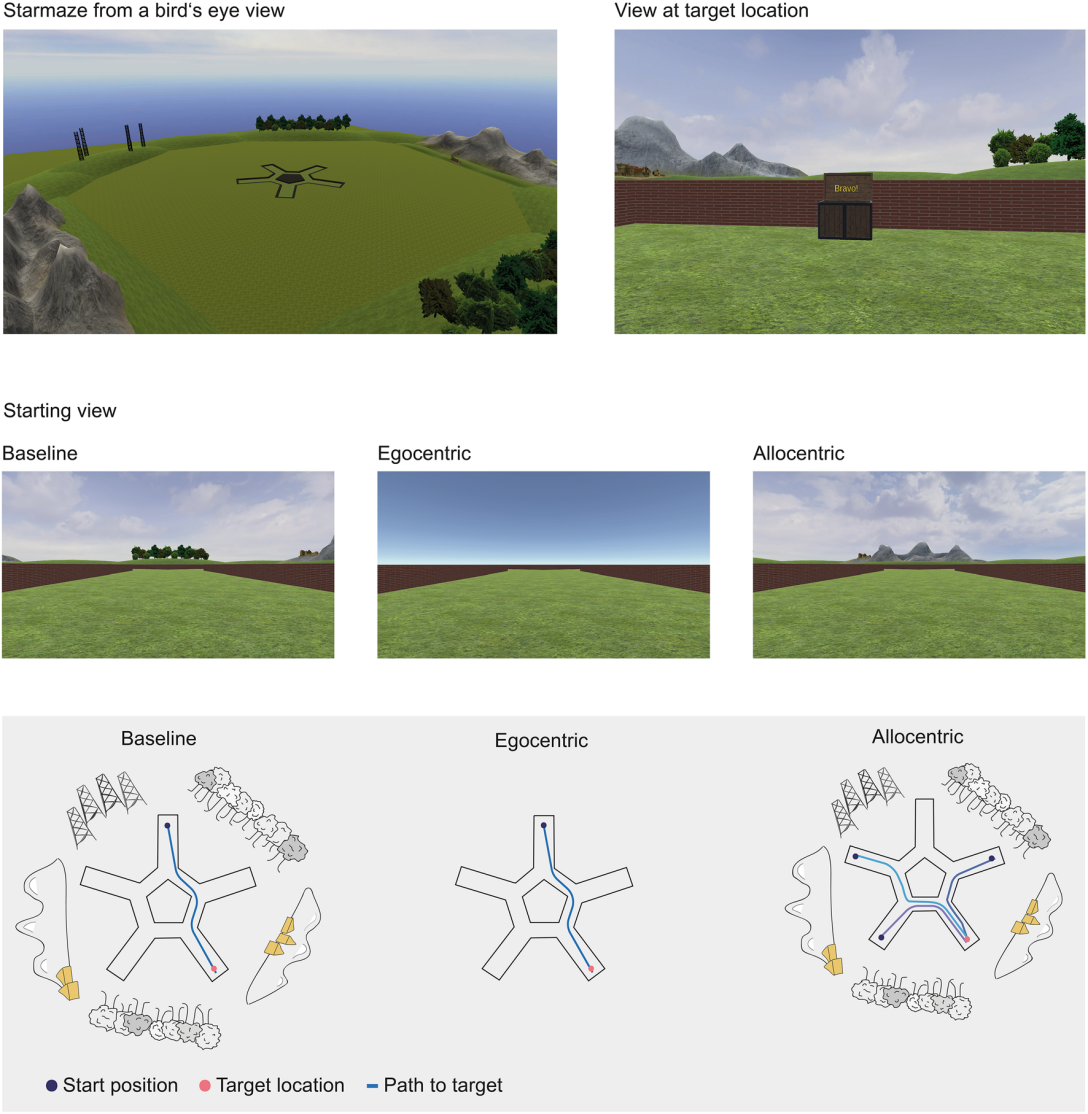
Virtual navigation task setup. First row, five-arm maze surrounded by environmental cues, bird’s eye view, and view at target location, participant’s view. Second row, example views at start location in baseline, egocentric and allocentric trials, participant’s view. Third row, schematic representation of the three types of conditions. Ideal paths depicted as blue lines connecting the start and target locations

Spatial navigation was tested with a virtual environment consisting of a five-armed star-shaped maze surrounded by environmental landmarks (Fig. 1). The maze was a modified version of the “starmaze” task derived from animal research [19–21, 31]. The maze consisted of five symmetrically arranged peripheral alleys connected by five central alleys and was surrounded by five distant environmental landmarks embedded in the virtual landscape. The task was implemented in Unity3D using the Unity Experiment Framework [32]. To create an immersive experience, we projected the screen on a white wall with a size of 140 × 80 cm at a distance of 200 cm. Participants used a joystick controller to navigate within the maze.

#### Memory-guided navigation task

To test whether motor abilities and joystick control were comparable between groups, we first asked participants to navigate to 15 sequentially appearing red balloons as quickly as possible. In the memory-guided navigation task, we then instructed participants to search for a hidden treasure in the virtual maze. The treasure was always in the same location and participants were asked to navigate directly to the treasure. Initially, the treasure appeared as soon as the subject reached its location (‘learning’ trials). We further informed participants that in some intermingled trials the treasure would not appear, even if the location was correctly remembered (‘probe’ trials). Instead, participants would have to indicate the memorized position by pressing a button at its location. All participants were informed that neither the maze nor the environment would change during the experiment. Trials were terminated four seconds after participants reached the target location in learning trials and after button-press in probe trials. If neither of these events occurred, the trial was terminated after 90 s.

The testing session consisted of three blocks with a total of 30 trials (details in Supplementary Table 1). During the first block, all landmarks were visible, and participants always started from the same starting point (‘baseline’ condition, Fig. 1). Participants could either use a strategy based on the remembered path sequences and body turns (‘egocentric’ strategy) or locate themselves relative to the distant landmarks (‘allocentric’ strategy) [19, 21]. In case participants did not find the treasure within the first three trials, a video showed them the shortest path to the target from a first-person perspective. In the second block, we removed all distant landmarks to force participants to use an egocentric strategy to locate the treasure (‘egocentric’ condition, Fig. 1). In the third block, the landmarks reappeared, but participants now started from novel starting positions. The participants were not immediately informed about this change but were debriefed about the change in starting position after the fourth trial. This manipulation was intended to force participants to use an allocentric strategy to locate the treasure (‘allocentric’ condition, Fig. 1).

After completion of the task, we asked participants to draw the spatial layout of the maze and the environmental landmarks from a bird’s eye perspective. Next, we showed them the correct maze layout and they had to indicate the position of the target location. We also asked participants to identify the correct five out of fifteen environmental landmarks and to position landmarks around the maze.

### Data pre-processing

While navigating the virtual maze, we recorded participant’s position as x- and y-coordinates in a Cartesian coordinate system. The coordinates were combined with a time stamp at a sampling rate of 60 Hz. Pre-processing of the data was performed in Matlab (Matlab 2020b, Mathworks, USA). We determined four parameters that were derived from animal studies and that represent different aspects of spatial navigation [21, 33, 34].

First, we determined whether our subjects successfully navigated to the correct location and calculated the percentage of successful trials for each subject for baseline, egocentric, and allocentric conditions (“success rate”). Second, for all successful probe trials, we determined the trial duration by subtracting the first from the last time stamp (“latency”). Third, we calculated the path error to the target location for successful probe trials, which reflects the directness of navigation (“path error to target”). Fourth, we calculated the average distance error to the target location for successful probe trials as a measure of search accuracy and as an expression of uncertainty in navigation behaviour (“search accuracy”). Additional details on the calculation of these variables can be found in the supplement.

Performance in the maze reconstruction task and landmark identification task was rated by three independent examiners according to predefined criteria. For quantifying the positioning of environmental landmarks, we used the Gardony Map Drawing Analyzer software [35]. As a result, we obtained three separate scores for maze reconstruction, landmark identification, and landmark positioning, ranging from zero to one with higher values denoting better performance. More details on the procedures can be found in the supplement.

### Statistical analysis

Statistical analyses were performed in RStudio (v. 3.5). To identify group differences of nominal variables, we used the *χ*^2^-independence test. We used a repeated measures ANOVA to assess group effects on latency, path error and search accuracy across baseline trials. In case of missing sphericity, a correction was applied. For between-group comparisons of the averaged probe trial data, we used non-parametric Wilcoxon rank sum tests because Shapiro–Wilk-tests showed that the assumption of normality had to be rejected for our main variables. Correlations between task performance and clinical markers of disease progression (ALS-FRS progression per month) and disease severity (ALS-FRS-R) were assessed using Spearman’s rank correlation coefficient. The level of significance was set to *p* < 0.05.

## Results

### Neuropsychological assessment

Seven out of 43 patients (16%) showed performance below ECAS cut-off values in domains relevant for Strong criteria of ALSci [24], i.e. verbal fluency (*n* = 1), executive function (*n* = 3) or language (*n* = 3). However, also in the control group, seven out of 43 subjects showed performance below ECAS cut-off values in executive function (*n* = 3) or language (*n* = 4). None of the participants had impairments in more than one domain. Across groups, we found no significant differences for executive function (*W* = 979.5, *p* = 0.502), verbal fluency (*W* = 1003, *p* = 0.266), language (*W* = 1019.5, *p* = 0.131) and visuospatial abilities (*W* = 927.5, *p* = 0.761) (descriptive values in Supplementary Table 3). ALS patients showed slightly inferior performance in verbal memory only (*W* = 1132.5, *p* = 0.043, effect size = 0.220). Moreover, ALS patients and healthy controls showed comparable performance in visuospatial fluency (5PT number of unique figures: *W* = 1037.5, *p* = 0.238), visuospatial memory (SPART immediate and delayed recall: *W* = 867.5, *p* = 0.758), and spatial orientation (PTSOT mean angle deviation: *W* = 882.5, *p* = 0.861).

### Memory-guided navigation

#### Hand motor function

Patients and controls did not differ with respect to joystick control abilities as indicated by comparable latency (*W* = 903, *p* = 0.857), path length (*W* = 866, *p* = 0.618) and velocity (*W* = 1001, *p* = 0.514) in the practise task.

#### Spatial learning in baseline condition

**Fig. 2:**
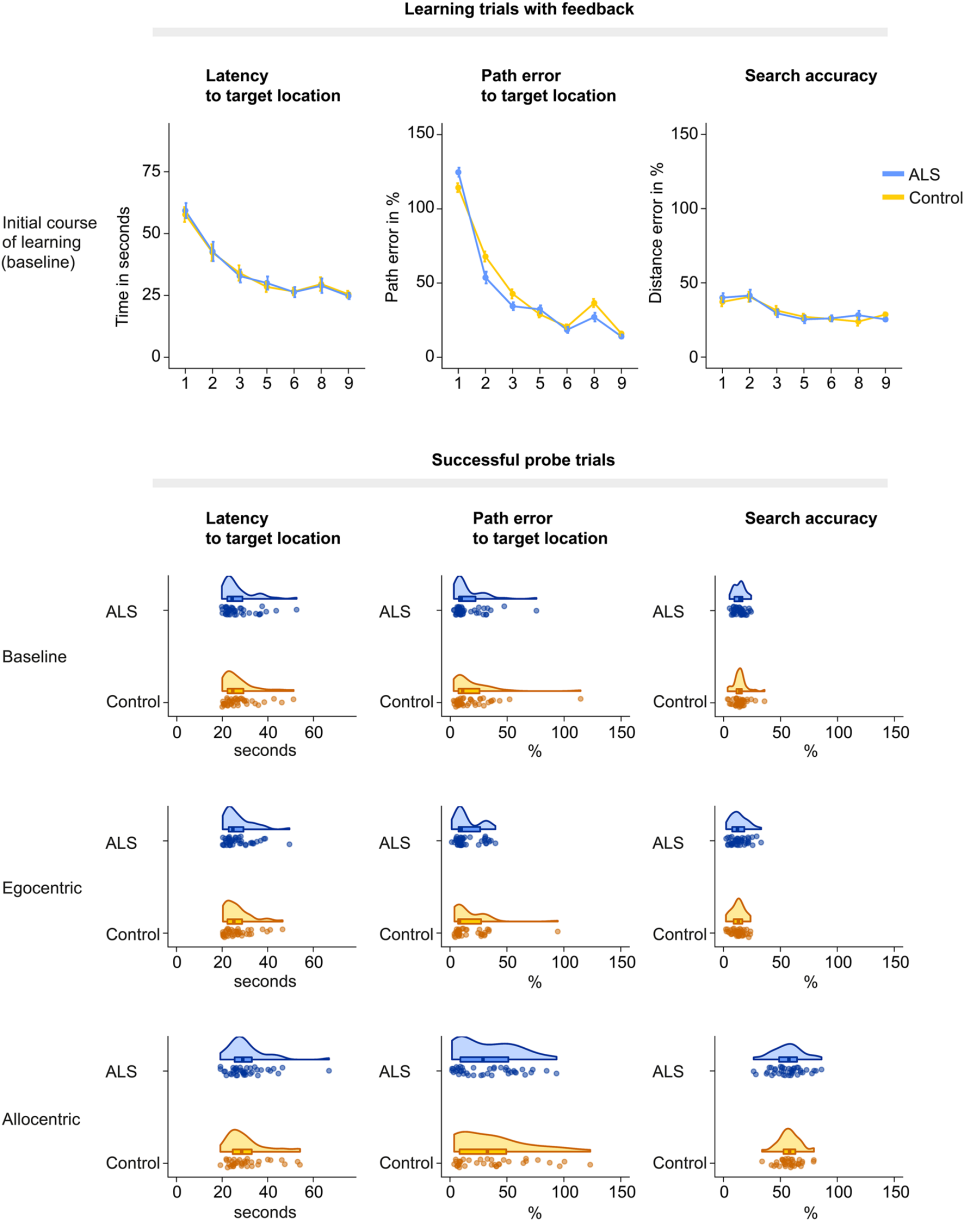
Performance in the starmaze task. Blue, ALS group; yellow, control group. First row, initial course of learning across trials in baseline condition. Second to fourth row, navigation performance in successful probe trials. Second row, baseline condition; third row, egocentric condition; fourth row, allocentric condition. Note that both groups show comparable spatial learning abilities and memory-guided spatial navigation performance, as indicated by the lack of group differences in latency, path error, and search accuracy. Data are presented as line plots with standard error and rain cloud plots with individual data points for each participant

Both groups showed comparable spatial learning abilities as indicated by a similar decrease in latency (repeated-measures ANOVA with Greenhouse–Geisser correction: *F*(3.814, 316.536) = 0.6356, *p* = 0.891), path error (repeated-measures ANOVA with Greenhouse–Geisser correction: *F*(4.091, 339.541) = 0.682, *p* = 0.574), and search accuracy (repeated-measures ANOVA with Huynh–Feldt correction: *F*(5.148, 427.284) = 0.803, *p* = 0.222) across baseline learning trials. Consistent with this observation, the success rate in baseline probe trials did not differ significantly between ALS patients (92.2%) and healthy controls (86.4%) (*W* = 773.5, *p* = 0.101). Patients also showed comparable navigation efficiency, as evidenced by similar latency (*W* = 940, *p* = 0.898), path error (*W* = 989, *p* = 0.582), and search accuracy (*W* = 926, *p* = 0.993) in successful baseline probe trials (Fig. 2).

#### Egocentric spatial navigation

Across all egocentric probe trials, 96.5% of ALS patients and 94.0% of healthy controls (*W* = 898.5, *p* = 0.937) were able to repeat the correct path sequence and successfully navigated to the correct target location. Navigation efficiency in successful egocentric probe trials was comparable between groups, with similar latency (*W* = 819, *p* = 0.714), path errors (*W* = 793, *p* = 0.546), and search accuracy (*W* = 846, *p* = 0.903) (Fig. 2, Supplementary Fig. 1, Supplementary Table 2). ALS patients thus acquired and used egocentric memory representations as efficiently as healthy controls.

#### Allocentric spatial navigation

Across all allocentric probe trials, 50.7% of ALS patients and 47.7% of healthy controls applied an allocentric strategy and successfully navigated to the target location (*W* = 872, *p* = 0.786). Latency (*W* = 692, *p* = 0.796), path error (*W* = 751, *p* = 0.732), and search accuracy (*W* = 770, *p* = 0.590) in successful allocentric probe trials were also comparable between patients and controls (Fig. 2, Supplementary Fig. 1, Supplementary Table 2). ALS patients thus acquired and used allocentric memory representations as efficiently as healthy controls.

#### Correlations between memory-guided navigation and clinical variables

We found no correlation of the success rate in the starmaze with disease severity (baseline: *r*_*s*_ = − 0.069, *p* = 0.662, egocentric: *r*_*s*_ = − 0.163, *p* = 0.297, allocentric: *r*_*s*_ = − 0.080, *p* = 0.608) or disease progression (baseline: *r*_*s*_ = − 0.146, *p* = 0.350, egocentric: *r*_*s*_ = 0.074, *p* = 0.639, allocentric: *r*_*s*_ = − 0.192, *p* = 0.218). Similarly, we found no association between navigation parameters of successful trials and disease severity (baseline: latency, *r*_*s*_ = − 0.201, *p* = 0.197; path error, *r*_*s*_ = 0.272, *p* = 0.171; search accuracy, *r*_*s*_ = 0.237, *p* = 0.126; egocentric: latency, *r*_*s*_ = − 0.213, *p* = 0.170; path error, *r*_*s*_ = 0.145, *p* = 0.354; search accuracy, *r*_*s*_ = 0.119, *p* = 0.449; allocentric: latency, *r*_*s*_ = − 0.255, *p* = 0.108; path error, *r*_*s*_ = − 0.006, *p* = 0.970; search accuracy, *r*_*s*_ = − 0.001, *p* = 1.0). For disease progression, we found a correlation only for latency across conditions, but not for other navigation parameters (baseline: latency, *r*_*s*_ = 0.544, *p* < 0.001; path error, *r*_*s*_ = 0.091, *p* = 0.563; search accuracy, *r*_*s*_ = 0.033, *p* = 0.835; egocentric: latency, *r*_*s*_ = 0.483, *p* = 0.001; path error, *r*_*s*_ = 0.041, *p* = 0.792; distance error, *r*_*s*_ = 0.010, *p* = 0.951; allocentric: latency, *r*_*s*_ = 0.310, *p* = 0.049; path error, *r*_*s*_ = − 0.109, *p* = 0.497; search accuracy, *r*_*s*_ = 0.016, *p* = 0.923).

We found no significant differences for any of the investigated navigational variables when ALS patients with normal ECAS values, ALS patients performing below ECAS cut-off values in domains relevant for Strong criteria of ALSci [24], controls with normal ECAS values and controls performing below ECAS cut-off scores were compared (all *p* ≥ 0.134, see Supplementary Table 4 for details). Furthermore, we found no significant differences for any of the investigated navigational variables when ALS patients, PMA patients, PLS patients and controls were compared (all *p* ≥ 0.084, see Supplementary Table 5 for details).

### Post-navigational memory for maze reconstruction, landmark identity, and landmark positioning

There were no significant differences between ALS patients and healthy controls in reconstruction of the maze layout (*W* = 924.5, *p* = 1), recall and identification of environmental landmarks (*W* = 991, *p* = 0.568), and positioning of landmarks on the map (*W* = 1030, *p* = 0.364).

## Discussion

To the best of our knowledge, this is the first study of spatial navigation in ALS. We investigated navigation in non-demented early- to mid-stage ALS patients and matched controls. We used a virtual reality task that simulates an important aspect of everyday behaviour and that has been shown to depend on integrity of hippocampus-dependent networks [15, 20]. Our study aimed to resolve conflicting findings on behavioural correlates of hippocampal alterations in ALS [5, 10, 36]. We found that ALS patients successfully learned and navigated a maze from memory, both in conditions that forced the use of landmark memory and memory of previously travelled path sequences. Memory-guided navigation success did not correlate with disease severity and progression. Measures of navigational efficacy as well as neuropsychological tests of spatial memory, visuospatial fluency and spatial orientation did not differ between patients and controls. We thus found no behavioural evidence for functionally relevant hippocampal dysfunction in our cohort of ALS patients.

Our study focussed on early to mid-stage ALS patients without evidence of frontotemporal dementia or behavioural symptoms and was restricted to ALS patients with preserved hand-motor function. Despite these limitations, our cohort closely matches previous studies in disease subtypes, disease duration, severity, and patient demographics. These studies reported several subtle abnormalities of medial temporal lobe structures, including hippocampal volume reductions, CA1 shape deformations, altered hippocampal functional connectivity and thinning of the parahippocampal gyrus [10, 36, 37]. These regions are essential nodes in networks for spatial navigation and memory [15]. Accordingly, deficits in spatial navigation were shown to be behavioural markers of hippocampal dysfunction in other neurodegenerative disorders [18]. To assess memory-guided navigation in ALS, we used an established navigation paradigm that is sensitive to hippocampal damage in navigating rodents [31], drives hippocampal activation in navigating humans [20] and captures changes in hippocampal function during human brain development, ageing and neurodegeneration [19, 38]. An important feature is that the paradigm allows the investigation of two navigation strategies (i.e. egocentric and allocentric), reflecting neural computations in distinct hippocampus-dependent memory networks [17]. We reasoned that changes in functional status of these networks should yield measurable changes of corresponding navigation variables in ALS patients. However, behaviour was indistinguishable from healthy controls, suggesting functional integrity of brain networks recruited during navigation.

Although motor symptoms are prominent in ALS patients, deficits of language, social cognition, executive functions and memory have been reported, even in patients that do not meet FTD diagnostic criteria [2–4]. Whether these cognitive deficits emerge early and remain stable [10, 39], decline as the disease progresses [40, 41] or emerge in later disease stages [4, 7] is still unclear. Facing the considerable overlap of ALS with FTD in genetic risk factors, neuropathology and brain atrophy profiles, it has been suggested that ALS and FTD may represent a continuous spectrum of clinical phenotypes [6]. One prediction of this hypothesis is that cognitive deficits, including those that relate to hippocampal dysfunction, are likely to emerge at some point during the disease course even in ‘pure’ ALS patients – provided they do not die because of motor impairment. One argument in favour of this hypothesis may be the observation of verbal memory impairment in non-demented ALS patients [9, 10]. A small but significant group difference for verbal memory was also observed in our sample. However, neuropathological studies show that verbal memory deficits do even occur in ALS patients without any hippocampal pathology [42]. It has thus been hypothesized that these deficits may relate to extra-hippocampal dysfunction, at least in cognitively otherwise normal ALS patients [11, 42].

While there is evidence that hippocampal structural pathology can occur in non-demented ALS patients, it is unclear if hippocampal dysfunction is limited to distinct ALS subpopulations or a general feature of ALS within a continuous ALS-to-FTD spectrum. A recent large study compared patients with pure ALS, i.e. without obvious cognitive or behavioural impairment, to ALS-FTD patients and controls [5]. Patients with ALS-FTD showed lower hippocampal volumes and verbal memory impairment compared to pure ALS patients and healthy controls. In contrast, hippocampal volume and memory in ALS-pure patients did not differ from healthy controls. Facing the heterogeneity of the neurodegenerative profiles, the authors concluded that ALS and FTD are unlikely to be variants of the same condition but may rather represent distinct syndromes with different brain atrophy profiles [5]. Our finding of intact spatial cognition in a sample of patients with an established diagnosis of ALS and an average disease duration that corresponds to the patients investigated in this study fits this hypothesis.

A limitation of our study is the lack of structural imaging data. We can thus not exclude that structural hippocampal abnormalities were present in some patients but did not yield detectable behavioral differences. The lack of longitudinal data further leaves open the possibility that distinct disease trajectories of ALS motor and cognitive symptoms account for our findings and that hippocampus-dependent cognitive deficits may occur in later disease stages [7, 43]. Since we found no correlations between spatial memory and disease severity or progression, we deem this explanation unlikely. It is possible, though, that cognitive reserve may have attenuated deficits in a context where hippocampal recruitment usually occurs [14]. Future studies may thus extend our approach to ALS-patients with more severe physical and/or cognitive impairments (e.g., executive dysfunction) and more aggressive disease progression.

## Conclusions

ALS patients without signs of FTD represent the majority of ALS patients and account for 85–95% of all ALS patients [1, 2]. Normality of navigational parameters and indices of visuospatial cognition in our cohort of early- to mid-disease stage ALS patients supports the emerging picture of distinct profiles for ALS and ALS-FTD in atrophy patterns and network impairments [5, 44]. Clinical care in ALS may therefore deliberately focus on individual affected networks rather than on a spectrum of possible disease trajectories in patients with intact cognition but impaired motor function and vice versa. Future studies should combine behavioural tasks with functional brain imaging to further investigate the relationship between spatial navigation and hippocampal pathology at different disease stages and in different ALS subtypes.

## Supplementary Information

Below is the link to the electronic supplementary material.

Supplementary file1 (PDF 976 KB)

## Data Availability

All analysis scripts and data are available on the Open Science Framework (osf): https://osf.io/s5crn/.
