# Supplementary material for: Memory-guided navigation in amyotrophic lateral sclerosis
Source: J Neurol. 2023 May 8;270(8):4031–40. doi: 10.1007/s00415-023-11753-8 (PMC10344997; doi:10.1007/s00415-023-11753-8)
Supplement: Supplementary file 1 — Supplementary file1 (PDF 976 KB) [file 415_2023_11753_MOESM1_ESM.pdf]

SUPPLEMENT

Memory-guided navigation in amyotrophic lateral sclerosis

Patrizia M. Maier[1,2]*, Deetje Iggena[1,2]*, Thomas Meyer[1], Carsten Finke[1,2],
Christoph J. Ploner[1]

Supplementary table 1. Block, conditions and trial order. *L* denotes learning trials, where the goal appeared at the target location. *P* denotes probe trial, where the goal did not appear and the participant had to press a button at the chosen target location. *Video* was only presented if the participant did not find the goal during the first three trials. In baseline trials, environmental landmarks were visible and participants started from the same starting point. In egocentric trials, environmental landmarks were invisible and participants started from the same starting point. In allocentric trials, environmental landmarks were visible and participants started from a new starting point.

Block	Condition	Trial order	Number of trials
1	baseline	*L – L – L – (L_{Video}) – L – L – P – L – L – P*	10
2	egocentric	*P – L – L - P*	4
3	allocentric	*P – L – P – P – P – L – L – L – L – L – L – L – L – P – P– P*	16

Supplementary material on data pre-processing

Success rate: We determined whether our subjects successfully navigated to the correct location in probe trials, by calculating the Euclidean distance between the target and the participant's final position.

$$\text{Euclidean distance} = \sqrt{\left(x_{target} - x_n\right)^2 + \left(y_{target} - y_n\right)^2}$$

We classified a trial as successful if the Euclidean distance was less than one-third of the length of an external maze alley. We then calculated the percentage of successful trials.

Latency: For successful probe trials, we calculated the trial duration by subtracting the first timestamp from the last timestamp.

$$\text{Latency} = t_n - t_1$$

Path error: For successful probe trials, we determined the total distance travelled by calculating both the participant's path length and the ideal path length to the target location. This measure reflects the degree of directness of navigation. We normalized the path length by calculating the absolute percent error.

$$\text{(Ideal) path length} = \sum_{i=1}^{n} \sqrt{(x_i - x_{i+1})^2 + (y_i - y_{i+1})^2}$$

$$\text{Path error} = \frac{(path_{ideal} - path)}{path_{ideal}} \times 100$$

Search accuracy: For successful probe trials, we determined the average distance to the target across all time stamps of a trial to determine how close to the target participants searched. This measure does not correlate with path length per se, but rather refers to the degree of uncertainty in navigation behaviour, even on trials with normal path length. We normalized the distance to target by calculating the absolute percent error.

$$\text{(Ideal) distance to target} = \frac{\sum_{i=1}^{n} \sqrt{\left(x_i - x_{target}\right)^2 + \left(y_i - y_{target}\right)^2}}{n}$$

$$\text{Search accuracy} = \frac{(distance_{ideal} - distance)}{distance_{ideal}} \times 100$$

Ratings of post-navigational memory

The maze reconstruction task and landmark identification task was rated by three independent examiners according to predefined criteria. Criteria for maze reconstruction: 1. evaluation of outer shape (1 point each): symmetric, closed figure, intersections with at least 2 choice points, single alley as connector between intersections, equal alley length, extra point for correct outer shape; 2. evaluation of inner shape (1 point each): singular structure, closed, squared, has 5 edges; 3. number of peripheral alleys (1 point each, minus if more than 5 alleys). The points for all three aspects were added and normalized to a score from zero to one; higher score denotes more accurate maze reconstruction. Criteria for landmark identification: Number of landmarks (1 point each, minus if more than 5 landmarks), accuracy of semantic categories of landmarks (1 point each, minus for category duplicates), 3. level of detail (1 point each): 4 electrical towers, mountain village, second mountain village. The points for all three aspects were added and normalized to a score from zero to one; higher

score denotes better landmark memory. To obtain final scores for each subject, the three examiner ratings were averaged.

For quantifying the positioning of environmental landmarks, we used the Gardony Map Drawing Analyzer software [34]. As recommended by the authors, we computed the SQRT(CanOrg) score, by determining pairwise canonical relations (north-south/east-west) between objects in the sketch map and comparing them to the target map. The SQRT(CanOrg) score ranges between zero and one with higher values denoting better overall configural accuracy and completeness.

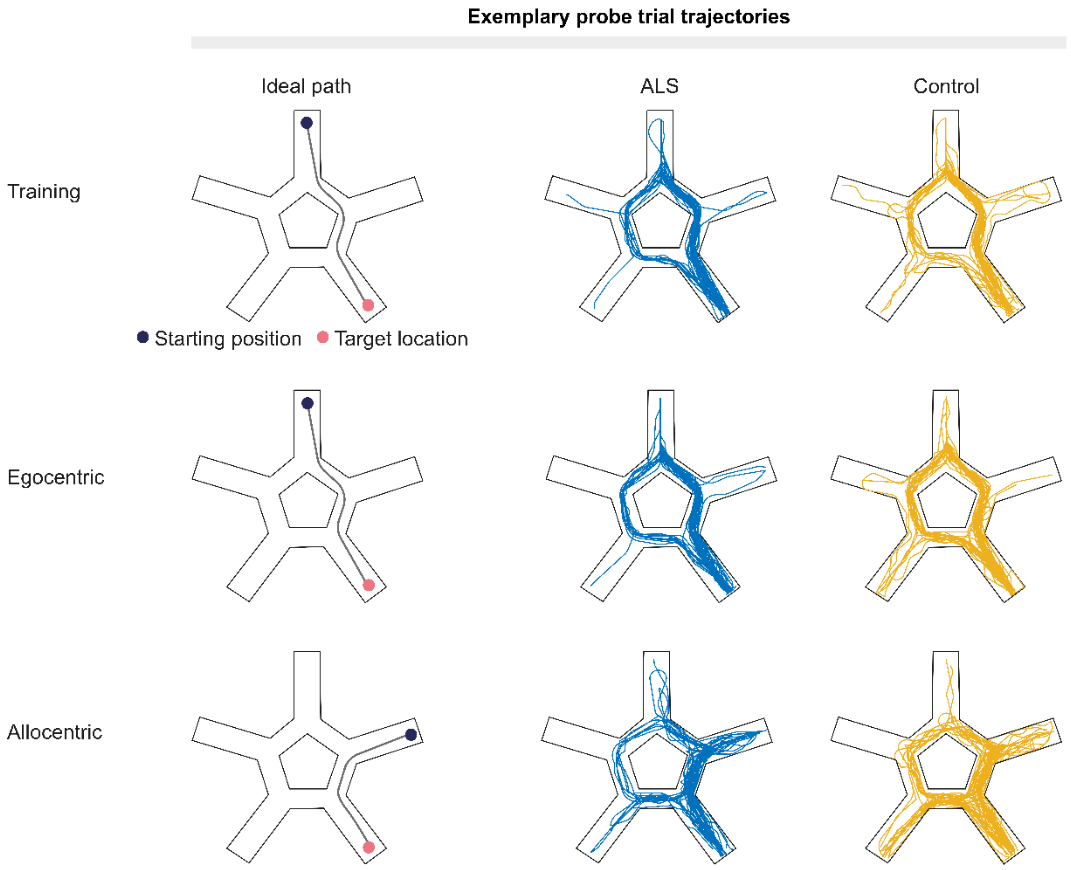

Supplementary figure 1. Example trajectories in selected probe trials. First row, baseline condition, second row egocentric condition, third row allocentric condition. The first column shows the ideal path from the start to the target, followed by the actual trajectories of the ALS group (blue), followed by the actual trajectories of the control group (yellow).

Supplementary table 2. Navigation results. Data presented as median with interquartile range (25 – 75%). Statistical analysis with Wilcoxon rank sum test. Abbreviations: ALS = amyotrophic lateral sclerosis.

	**ALS** (n = 43)	**Control** (n = 43)	***p-value***
**Baseline (successful probe trials)**			
Latency to target location (seconds)	24.28 (22.14 – 28.29)	24.58 (22.24 – 29.25)	p = 0.898
Path error to target location (%)	9.59 (7.62 – 22.22)	10.90 (7.22 – 25.71)	p = 0.582
Search accuracy (%)	14.29 (9.45 – 16.60)	14.13 (11.28 – 16.07)	p = 0.993
**Egocentric (successful probe trials)**			
Latency to target location (seconds)	24.75 (22.87 – 29.35)	25.14 (22.31 – 28.79)	p = 0.714
Path error to target location (%)	9.84 (7.56 – 26.46)	8.38 (7.09 – 27.32)	p = 0.546
Search accuracy (%)	12.38 (7.78 – 18.37)	13.23 (9.0 – 16.68)	p = 0.903
**Allocentric (successful probe trials)**			
Latency to target location (seconds)	29.10 (25.49 – 33.05)	28.58 (24.64 – 33.03)	p = 0.796
Path error to target location (%)	29.02 (9.12 – 51.31)	32.88 (8.43 – 49.44)	p = 0.732
Search accuracy (%)	57.51 (48.87 – 64.65)	58.09 (52.74 – 63.17)	p = 0.590
**Post-navigation memory**			
Landmark identity	0.79 (0.71 – 0.86)	0.81 (0.75 – 0.85)	p = 0.568
Landmark position	0.80 (0.71 – 0.86)	0.82 (0.75 – 0.88)	p = 0.364
Maze reconstruction	0.69 (0.50 – 0.81)	0.73 (0.44 – 0.78)	p = 1.000

Supplementary table 3. Neuropsychological results. Data presented as median with interquartile range (25 – 75%). Statistical analysis with Wilcoxon rank sum test. Abbreviations: ALS = amyotrophic lateral sclerosis; ECAS = Edinburgh Cognitive and Behavioural ALS Screen; SPART = 10/36 Spatial Recall Test; 5PT = Five Point Test; PTSOT = Perspective Taking/Spatial Orientation Test.

	**ALS** (n = 43)	**Control** (n = 43)	***p-value***
**ECAS**			
Executive	39.50 (38.00 – 41.75)	40.00 (38.00 – 43.00)	p = 0.502
Verbal fluency	20.00 (18.00 – 22.00)	20.00 (18.00 – 22.00)	p = 0.265
Language	27.00 (26.00 – 27.25)	27.00 (26.00 – 28.00)	p = 0.131
Verbal memory	18.00 (16.00 – 19.00)	19.00 (17.00 – 21.00)	p = 0.043
Visuospatial	12.00 (12.00 – 12.00)	12.00 (12.00 – 12.00)	p = 0.761
**SPART**			
Immediate memory	6.67 (5.67 – 7.92)	7.00 (5.67 – 7.67)	p = 0.902
Delayed memory	8.00 (6.00 – 9.00)	7.00 (6.00 – 9.00)	p = 0.398
Overall memory	7.00 (5.75 – 8.12)	6.75 (5.62 – 7.75)	p = 0.758
**5PT**			
Visuospatial fluency	22.50 (19.00 – 28.00)	24.00 (21.00 – 29.00)	p = 0.238
**PTSOT**			
Spatial orientation	41.19 (23.08 – 71.36)	43.33 (17.58 – 67.79)	p = 0.860

Supplementary table 4. Performance in the starmaze task for ALS patients with normal ECAS scores (ECAS+), ALS patients with ECAS scores below cut-off values relevant for Strong criteria (Strong et al., 2017) i.e. verbal fluency, executive function or language function (ECAS-), controls with normal ECAS scores (ECAS+), controls with ECAS scores below cut-off in fluency, executive or language function (ECAS-). Data presented as median with interquartile range (25 – 75%). Values for latency, path error and search accuracy based on successful probe trials. Statistical analysis with Kruskal-Wallis rank sum test. Abbreviations: ALS = amyotrophic lateral sclerosis; Ctrl = controls. Note comparable memory-guided spatial navigation performance for all investigated parameters across groups.

	**ALS-ECAS+** (n= 36)	**ALS-ECAS-** (n = 7)	**Ctrl-ECAS+** (n = 36)	**Ctrl-ECAS-** (n = 7)	***p-value***
**Baseline**					
Success rate	1.00 (1.0-1.0)	1.00 (0.8-1.0)	1.00 (0.7-1.0)	0.67 (0.6-1.0)	.134
Latency	24.40 (22.2-29.3)	23.92 (21.8-26.8)	25.72 (22.3-29.6)	22.98 (21.7-24.8)	.777
Path error	9.39 (7.7-18.9)	10.73 (6.8-29.0)	11.30 (7.3-25.5)	9.51 (7.6-20.5)	.895
Search accuracy	14.49 (9.1-16.5)	13.78 (12.2-16.1)	14.19 (11.3-16.1)	12.94 (11.7-17.9)	.994
**Egocentric**					
Success rate	1.00 (1.0-1.0)	1.00 (1.0-1.0)	1.00 (1.0-1.0)	1.00 (1.0-1.0)	.722
Latency	25.70 (22.9-30.3)	24.01 (22.4-26.3)	26.26 (22.8-29.0)	21.59 (20.5-24.1)	.265
Path error	9.78 (8.2-20.2)	14.12 (6.2-28.4)	9.38 (6.8-27.9)	8.08 (7.6-13.0)	.874
Search accuracy	12.80 (7.3-20.1)	11.61 (10.7-14.1)	13.40 (8.6-16.8)	12.31 (11.6-13.5)	.953
**Allocentric**					
Success rate	0.57 (0.3-0.7)	0.43 (0.4-0.4)	0.50 (0.4-0.7)	0.43 (0.1-0.5)	.186
Latency	29.58 (26.1-34.9)	26.48 (22.0-28.8)	28.67 (25.0-32.5)	24.37 (23.7-53.2)	.488
Path error	33.04 (9.6-52.5)	13.29 (7.0-32.8)	28.18 (8.1-46.2)	43.33 (40.9-100.6)	.311
Search accuracy	57.84 (50.5-65.4)	49.24 (44.7-59.6)	57.48 (52.1-62.0)	59.88 (54.5-69.3)	.406

Supplementary table 5. Performance in the starmaze task for patients with ALS, PLS, PMA, and controls. Data presented as median with interquartile range (25 – 75%). Values for latency, path error and search accuracy based on successful probe trials only. Statistical analysis with Kruskal-Wallis rank sum test. Abbreviations: ALS = amyotrophic lateral sclerosis; PLS = primary lateral sclerosis, PMA = progressive muscular atrophy. Note that all groups show comparable memory-guided spatial navigation performance, as indicated by the lack of group differences in success rate, latency, path error, and search accuracy.

	**ALS** (n = 32)	**PLS** (n = 4)	**PMA** (n = 7)	**Ctrl** (n = 43)	***p-value***
**Baseline**					
Success rate	1.00 (0.9-1.0)	1.00 (0.9-1.0)	1.00 (1.0-1.0)	1.00 (0.7-1.0)	.217
Latency	25.26 (23.2-30.6)	21.58 (20.9-23.7)	22.12 (21.7-22.3)	24.58 (22.2-29.2)	.084
Path error	9.80 (7.6-26.1)	7.83 (7.3-8.4)	10.55 (8.5-11.6)	10.90 (7.2-25.7)	.599
Search accuracy	15.40 (11.2-17.9)	9.94 (8.3-11.8)	11.03 (10.1-14.8)	14.13 (11.3-16.1)	.125
**Egocentric**					
Success rate	1.00 (1.0-1.0)	1.00 (1.0-1.0)	1.00 (1.0-1.0)	1.00 (1.0-1.0)	.792
Latency	26.09 (23.5-30.9)	21.65 (21.0-25.0)	23.12 (22.8-23.7)	25.14 (22.3-28.8)	.156
Path error	10.90 (7.8-30.5)	9.16 (8.2-9.3)	10.50 (7.9-11.0)	8.38 (7.1-27.3)	.637
Search accuracy	13.09 (7.8-21.4)	10.13 (7.2-12.7)	11.61 (8.8-15.6)	13.23 (9.0-16.7)	.599
**Allocentric**					
Success rate	0.43 (0.3-0.6)	0.50 (0.2-0.8)	0.71 (0.5-0.9)	0.46 (0.4-0.7)	.269
Latency	29.09 (25.7-33.7)	29.89 (27.4-32.8)	28.27 (25.9-30.0)	28.58 (24.6-33.0)	.982
Path error	27.38 (8.4-50.3)	36.70 (24.2-54.0)	33.04 (17.7-52.4)	32.88 (8.4-49.4)	.824
Search accuracy	57.40 (47.8-63.3)	60.44 (59.8-65.0)	51.34 (47.1-64.0)	58.09 (52.7-63.2)	.536
